# The Impact of a Physiotherapy Tele-Rehabilitation Program on the Quality of Care for Children with Juvenile Idiopathic Arthritis

**DOI:** 10.31138/mjr.310823.tio

**Published:** 2023-08-31

**Authors:** Maria Stavrakidou, Maria Trachana, Artemis Koutsonikoli, Kyriaki Spanidou, Alexandra Hristara-Papadopoulou

**Affiliations:** 1 Asklepeio Physiotherapy Clinic, Thessaloniki, Greece,; 2 First Department of Paediatrics, Paediatric Immunology and Rheumatology Referral Centre, Aristotle University of Thessaloniki, Hippokration Hospital, Thessaloniki, Greece,; 3 Department of Physiotherapy, International Hellenic University, Thessaloniki, Greece

**Keywords:** tele-rehabilitation, physiotherapy, Juvenile Idiopathic Arthritis, home exercise program

## Abstract

**Objectives:**

To investigate the applicability and impact of a physiotherapy tele-rehabilitation program (TRP) on children with Juvenile Idiopathic Arthritis (JIA) and their families.

**Methods:**

Thirty JIA patients, applying an individualized home-exercise program (HEP), were randomly divided in the tele-rehabilitation (TRG, n=15) and control group (CG, n=15). Each TRG patient participated in a 30-minute tele-session, under a paediatric physiotherapist’s supervision, twice a week, for 12 weeks. Before and after the TRP (T1 and T2, respectively), all participants and a parent/guardian completed the Juvenile Arthritis Multidimensional Assessment Report (JAMAR) questionnaire and a questionnaire regarding the HEP implementation and compliance. Residual disease was estimated at T1 and T2. At T2, TRG patients/parents completed a questionnaire evaluating the TRP. One month after T2, a reassessment of compliance with the HEP was performed.

**Results:**

The patients’ median age was 12.8 (8-16) years. At T2, the TRG patients performed the HEP significantly more frequently (p=0.023), for a longer time (p=0.034) and with less urging (p=0.004), compared to T1. Moreover, they exhibited significantly increased compliance with HEP (p=0.001), better functionality (p=0.008), better quality of life (p=0.007) and less pain (p=0.017). The CG patients showed no significant changes. Residual disease improved in both groups (TRG:p=0.002, CG:p=0.018), but more in the TRG (p=0.045). TRP’s applicability and total benefit were rated as excellent by patients/parents. Finally, one month after T2, compliance with the HEP was still greater than at T1(p=0.001).

**Conclusion:**

An interactive physiotherapy TRP can be implemented effectively for JIA patients, providing an additional tool for their rehabilitation.

## INTRODUCTION

The World Health Organization (WHO) defines Telemedicine Services as the use of information and communication technologies by health care professionals for disease diagnosis, treatment, and prevention as well as for health workers’ continuous education.^1^

A particular focus of telemedicine is the development of tele-recovery systems to facilitate at-home physiotherapy for patients. The Internet provides services available to everyone, at any time, irrespective of distance, which contribute to saving time, and eliminating travel costs. It also enables attractive interactions (feedback) and visual interactivity (video real-time meetings). ^2,3^

The precise content of physiotherapeutic tele-rehabilitation and the patients, in whom it is addressed, remain to be more clearly elucidated. The few studies so far, mainly involve adult patients with musculoskeletal or cardiorespiratory disorders.^4–9^

Tele-physiotherapy can be implemented in adult and paediatric Rheumatology.

As for Juvenile Idiopathic Arthritis (JIA) patients, the disease chronicity and complications affect, in the long run, the quality of life (QOL). This depends mostly or mainly on self-management, compliance to treatment and participation in programs promoting physical and emotional well-being.^10–13^ It is therefore necessary for health professionals to adopt effective and innovative ways to promote self-management efficiently.^14–18^ Physiotherapy for JIA patients involves individualized programs and education of the child and at least one parent or guardian for at-home practice.

The Greek health system reimburses only 2x10 physical therapy sessions, regardless of the course of the disease and the existence of residuals, and no related travel expenses. Number of sessions that rarely cover the annual need in most patients with JIA. This was the motivation for finding more economical methods of providing physical therapy.

In the present study, our main objective was to investigate the possibility of implementing a physiotherapy tele-rehabilitation program (TRP) in patients with JIA and its effects on patients/families (quality of life, pain, disease residues and compliance). As a secondary objective, we investigated the effects of the individualised program on characteristics related to the program of therapeutic exercises (frequency and time of implementation and need for incentives from parents to children to implement the program). Finally, the degree of satisfaction of patients and parents after the end of the intervention program was assessed.

## METHODOLOGY

### Selection Criteria

Thirty, randomly selected patients, followed up in a Paediatric Immunology and Rheumatology Referral Centre met the following entry criteria:

- Diagnosis of JIA by meeting the International League of Associations in Rheumatology (ILAR) 2001 criteria
- Age 8 to 16 years old
- Physician’s Global Assessment in a visual analogue scale (Vas) <2
- Inactive residues of the disease
- Fluency in Greek (patient and his/her family)
- Current practice of a home-exercise program, as part of their physiotherapeutic rehabilitation, according to the treatment group instructions
- Having the basic, necessary equipment for the study All patients and their parents were informed about the conduct of the study and all accepted.

### Description of the Study Process and Assessment Tools

Duration of study September 2017 to May 2018. The telerehabilitation program took place from 2/11/2017 to 21/2/2018.

All patients and at least one parent or guardian had the same training for their individualised home exercise program and received instructions on how to apply it at least 3 times a week. All participants completed a questionnaire for the overall assessment of their disease status, the Juvenile Arthritis Multidimensional Assessment Report (JAMAR) and a questionnaire -designed for this research- regarding physiotherapy and the home-exercise program. The JAMAR questionnaire is a special questionnaire for multidimensional (total) assessment of the effects of JIA on the physical, psychological, and social functioning, as perceived and recorded by the children themselves and their parents for ages 8 years and older and only parents for ages up to 8 years. It has been validated for the Greek population.^19,20^ Finally, patients and their parents are familiarised with its completion, as it is used in our Rheumatology Referral Centre since 2011. In order to quantify patients’ functionality, their responses to the 15 “functional capacity assessment” questions were scored as follows: no difficulty=3 points, little difficulty=2 points, very difficult=1 point, cannot be performed=0 degrees. Then, the scores of each response for each patient were summed, resulting in a sum with a maximum value of 45 (excellent functioning) and a minimum of 0. Accordingly, in order to quantify patients’ quality of life, their responses to the 10 “functional capacity assessment” questions were scored as follows: never=3 points, sometimes=2 points, often=1 point, every day=0 points. Then, the scores of each response for each patient were summed, resulting in a sum with a maximum value of 30 (excellent quality of life) and a minimum value of 0.

### New questionnaire

For the needs of this research, a new evaluation questionnaire was created for the patient and for the parent or guardian, in relation to the patient’s participation in a program of physical therapy sessions and in relation to the implementation of the exercise program at home. The assessment of the child’s compliance in the application of the exercise program at home, on a VAS type scale from 0–10.

All patients and their parents signed an information and consent form according to the Declaration of Helsinki and the study was approved by consent by the Ethics Committee 4/5/2018 second Annual Meeting of the International Hellenic University, Thessaloniki, Greece.

The 30 patients were randomly divided into two groups: 15 in the tele-rehabilitation group (TRG) and 15 in the control group (CG), and all continued the home exercise program. The control group was aware of participating in a study, with no knowledge of the intervention in the trial group.

The disease residues were recorded in both groups. In particular, the following parameters were measured: a. the free range of motion (ROM) of the previously affected but currently inactive joints, using a common goniometer and the neutral-zero method, b. muscular atrophy, and c. the maximum mouth opening, in cases with temporomandibular joint involvement and d. the motion of the lumbar spine with the Schober test. All measurements were performed before and immediately after the TRP (TRG patients), at study entry and 12 weeks later (CG patients), at the physiotherapy office or the outpatient clinic, by a physiotherapist separate from the one performing the TRP.

Preliminary video-sessions were held for each patient (altogether 21) after Skype was installed on their computer, necessary for the familiarisation with the process and equipment. Specific days and hours per week were set for each patient. In particularly, two video-sessions for each patient, of 30 minutes each, every week, for 12 consecutive weeks, were conducted by the same specialised paediatric physiotherapist. Specifically, it included exercises to improve range of motion, exercises to improve elasticity and exercises to improve proprioception. Ball, balance disc and low resistance rubber were used where necessary. The development in the program concerned the increase in contraction duration from 4” to 6” and the number of repetitions from 8 to 10. Finally, there was ergonomics training in the sitting position.

During the regular JIA patient’s follow-up of both groups, at least one physiotherapeutic and one medical re-evaluation were carried out. An increase in Physician’s Global Assessment due to JIA activity was the only chance of excluding the patient from the research, apart from their voluntary withdrawal. If patients participated in physical therapy sessions at the same time, its frequency or content was not altered.

The control group applied the individualised therapeutic exercise program at home (had received training) 3 times a week with the help of their parents.

The difference between the two groups is the person-alised supervision during the implementation of the exercise program, the continuous interaction during the process, with encouragement and corrections, the relief of the parents from the responsibility of the implementation of the program and all this in an environment that is attractive for these ages (internet) and familiar (in their home).

After the TRP completion (TRG patients) and 12 weeks after study entry (CG patients) the same questionnaires with the ones at the study entry were completed. For TRG patients, questions evaluating the TRP were included. Finally, one month after the TRP completion, one videoconference for each TRG patient was conducted, in order to assess patients and parents’ compliance with the home exercise program (Vas type scale from 0-10). A total of 396 tele-rehabilitation sessions took place (21 educational, 360 TRP and 15 re-evaluation sessions, one month after the end of TRP).

### Statistical analysis

Results were reported using the mean value and standard deviation for the normally distributed variables, while for non-normally distributed variables the median value and the range of values. To test the normality of continuous variables the Shapiro-Wilk test was used. For the comparison of quantitative variables between the two levels of a qualitative variable, the student’s t-test for independent samples in the case of normally distributed variables and the non-parametric Mann-Whitney test, in case of non-normally distributed variables, were used. For the comparison between qualitative variables, the x2 (chi-square) test was used and in cases with an expected number of frequencies <5, the Fisher’s exact test. For the comparison of paired qualitative data, the non-parametric Mc Nemar assay was used, while for the comparison between paired quantitative data, the non-parametric Wilcoxon signed rank test. The Wilcoxon one-sample sign-rank test was used to check the statistical significance of the percent change of the patients’ ROM.

The level of statistical significance was set as p≤0.05. For the statistical analysis of the data, the IBM SPSS Statistics 22 statistical package was used.

## RESULTS

The patients’ demographics in both study groups are shown in **Table 1** and the changes regarding the implementation of the home exercise program at the end of the study, in the 2 groups of patients and controls, in **Table 2**.

**Table 1.** Demographics of all study patients and data regarding Juvenile Idiopathic Arthritis.

**Parameter**	**Total patients (n=30)**	**Tele-rehabilitation group (TRG) (n=15)**	**Control group (CG) (n=15)**	**p^1^**

**Age (years)^2^**	12.8 (8–16)	12 (8–16)	13 (8–16)	ns

**Residence ^3^**	Village: 9 (30)	Village: 8 (53)	Village: 1 (7)	0.014
	City: 21 (70)	City: 7 (47)	City: 14 (93)	

**Parents’ age (years)^4^**	43.5 ± 5.7	41.3 ± 5.2	45.7 ± 5.4	0.031

**Parents’ employment^3^**	Yes: 20 (67)	Yes: 10 (67)	Yes: 10 (67)	ns
	No: 10 (33)	No: 5 (33)	No: 5 (33)	

**Parents’ education level^3^**	High: 20 (67)	High: 12 (80)	High: 8 (53)	ns
	Middle: 10 (33)	Middle: 3 (20)	Middle: 7 (47)	

**JIA type^3^**	Polyarticular: 19 (63)	Polyarticular: 8 (53)	Polyarticular: 11 (73)	ns
	Oligoarticular: 11 (37)	Oligoarticular: 7 (47)	Oligoarticular: 4 (27)	

1 The p refers to comparisons between the 2 groups of patients;

2 Median (range);

3 Number of patients (%);

4 mean value ± standard deviation; JIA: Juvenile Idiopathic Arthritis; ns: not significant.

**Table 2.** Changes in quality parameters at the end of the study in the two groups of patients.

**Parameter**	**TRG [number of patients (%)]**	**CG [number of patients (%)]**	**p**
**Increase in frequency of HEP implementation**	6 (40)	0	0.024
**Increase in duration of HEP implementation (children’s responses)**	5 (33)	5 (33)	ns
**Increase in duration of HEP implementation (parents’ responses)**	5 (33)	0	0.042
**No need for urging to perform HEP^1^**	5 (38)^2^	0^3^	0.041
**Reduction in advice to perform HEP (children’ responses)**	10 (67)	0	<0.001
**Reduction in advice to perform HEP (parents’ responses)**	8 (53)	6 (40)	ns

1 Responses to this question were given by parents;

2 Of the 13 patients who needed encouragement, at the beginning of the study;

3 Of the 11 patients who needed encouragement, at the beginning of the study; TRG: tele-rehabilitation group, CG: control group; ns: not significant; HEP: home-exercise program.

### Compliance with the home-exercise program

**Figure 1.**
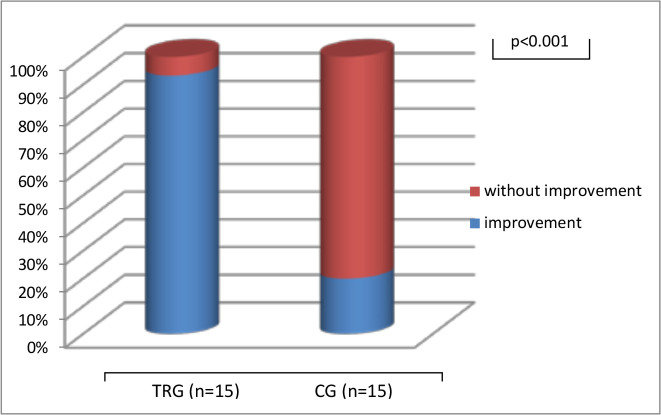
Improvement of compliance with the home exercise program in the two groups of patients (TRG: tele-rehabilitation group, CG: control group) at the end of the study.

A total of 14/15 patients (93%) of the TRG and 3/15 (20%) patients of the CG reported improved compliance. The difference between the two groups was statistically significant (p <0.001). (**Figure 1**)

**Figure 2.**
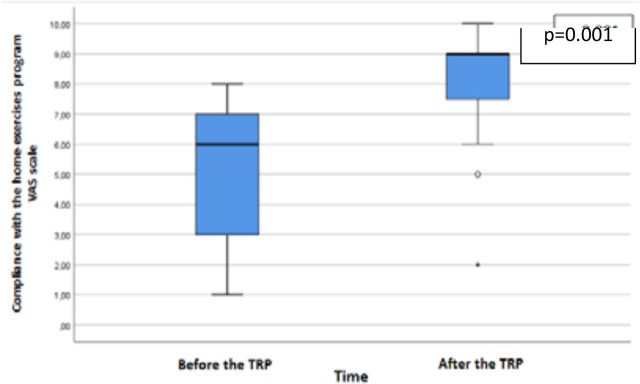
Compliance of the TRG (tele-rehabilitation group) patients with the home exercise program before and after the tele-rehabilitation program (TRP).

The compliance of the TRG patients with the home exercise program before and after the TRP is shown in **Figure 2**.

### Limited ROM

TRG: Before the TRP, 12/15 patients showed reduced ROM in at least one joint. In all patients, the affected joints’ ROM improved after the TRP. The median improvement of ROM per patient was 21.59 (13.22-287.61) % (p = 0.002).

CG: At the start of the study, 7/15 patients had reduced ROM in at least one joint. In all patients, the affected joints’ ROM improved at the end of the study. The median improvement of ROM per patient was 12.8 (8.33-20.17) % (p = 0.018).

**Figure 3.**
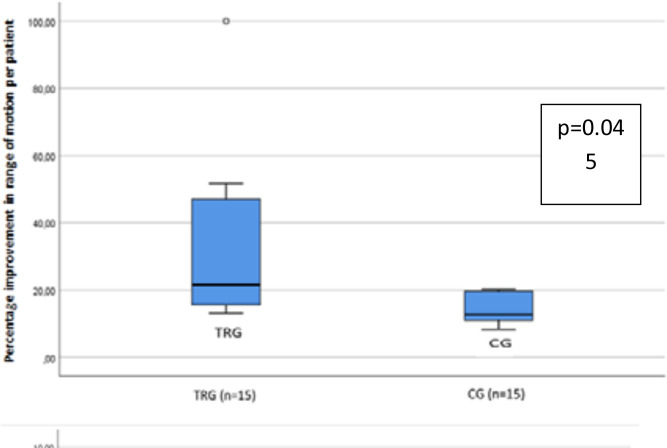
Percentage improvement in range of motion per patient in the two groups of patients (TRG: tele-rehabilitation group, CG: control group) at the end of the study.

Comparing the 2 groups, this improvement was statistically significantly greater in the TRG (p = 0.045). (**Figure 3**)

### Schober test

TRG: Before the TRP, the 15 patients’ median Schober score was 13.7 (12.8 to 15.6). After the TRP, a statistically significant increase was observed: 15 (14,5-17) (p = 0.001).

CG: At the beginning of the study, the 15 patients’ median Schober score was 14 (12.6-15). At the end of the study, a statistically significant increase was observed: 14.4 (13.2-15.8) (p = 0.001).

Among the 13 TRG patients with an abnormal Schober test before the TRP, 7 (54%) had a normal Schober test after TRP. In the CG, among the 14 patients with an abnormal Schober test at the beginning of the study, 4 (29%) had a normal Schober test at the end of the study. The difference between the two groups was not statistically significant.

### Pain

**Figure 4.**
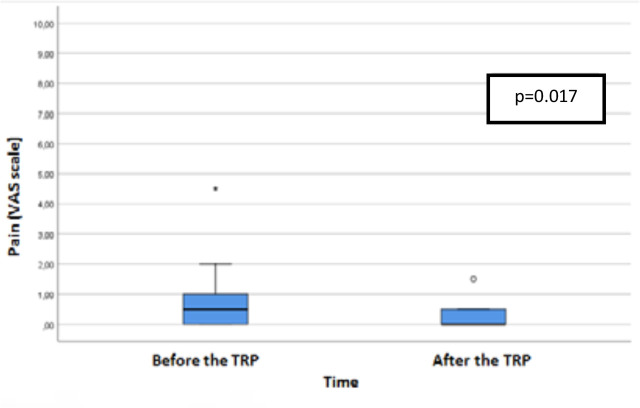
Estimation of pain in the TRG (tele-rehabilitation group) patients before and after the tele-rehabilitation program (TRP).

TRG: Before the TRP, the patients rated their pain on a VAS scale, with a median value 0.5 (0-4.5). After the TRP, the corresponding value was significantly lower: 0 (0-1.5) (p = 0.017) (**Figure 4**).

CG: At the beginning of the study, the patients’ pain on a VAS scale had a median of 0 (0-2.5). At the end of the study, the corresponding value did not differ statistically significantly: 0 (0-2).

Overall, 7/8 (88%) TRP patients who reported pain before the program, experienced pain relief after the program.

Meanwhile in the CG, in 2/6 patients (33%) who reported pain at the beginning of the study, the pain was reduced at the end of the study. The number of patients who reported pain at the beginning of the study was small to compare between the two groups.

### Quality of life (QOL)

**Figure 5.**
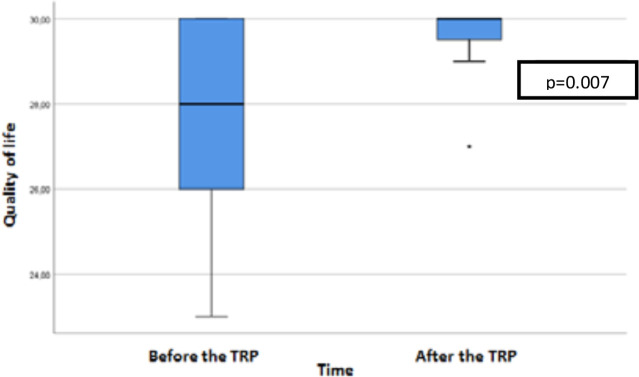
Quality of life of the in the TRG (tele-rehabilitation group) patients before and after the tele-rehabilitation program (TRP). Quality of life is measured on a 0-30 scale, where 30 corresponds to the best quality of life, as per the relevant domain of the Juvenile Arthritis Multi-dimensional Assessment Report (JAMAR) questionnaire.

**Figure 6.**
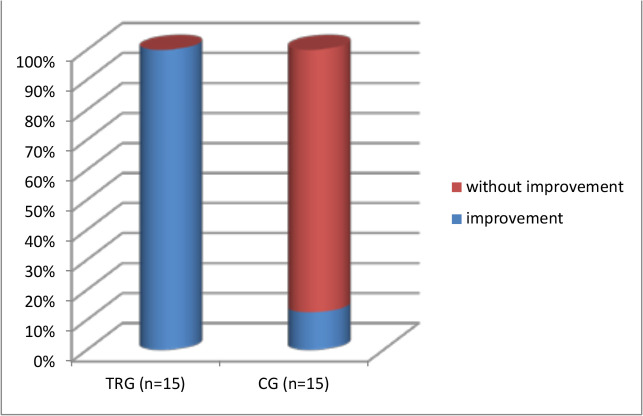
Improvement of the quality of life in the two groups of patients (TRG: tele-rehabilitation group, CG: control group) at the end of the study.

In the TRG, in all 9 patients with affected QOL before the TRP, QOL improved after the TRP (**Figure 5**). In contrast, in the CG, out of the 8 patients with affected QOL at the beginning of the study, the QOL improved only in 1 (12.5%) at the end. This difference between the 2 groups was statistically significant (p <0.001). (**Figure 6**)

### Re-evaluation of the TRG patients 1 month after the end of the program

The compliance to the home-exercise program according to the 15 patients’ responses was rated with a median of 8 (4-10). It did not differ from the corresponding value immediately after the TRP, while compliance continued to be statistically significantly higher than that before the TRP (p = 0.001).

According to parents’ responses, patient compliance to the exercise program was rated with a median of 6 (2-9).

Parents’ responses differed significantly from those of the children (p = 0.001).

The physiotherapist’s assessment of the competence in the exercises and the patient’s mode of application was scored on a VAS scale with a median of 10 (8-10).

**Figure 7.**
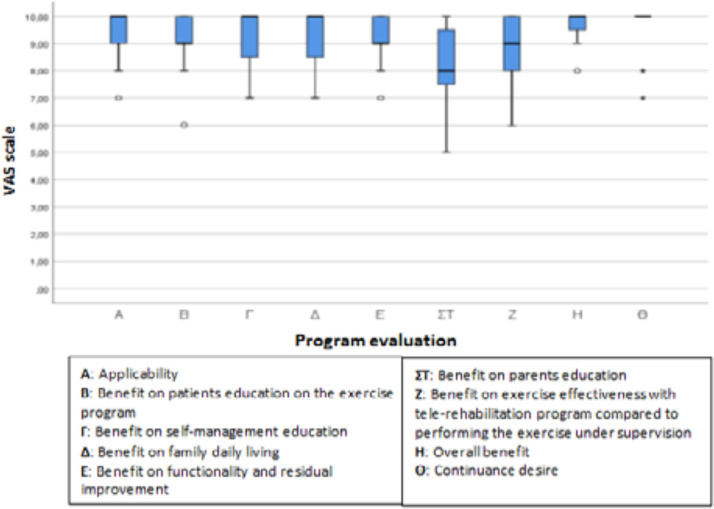
Evaluation of the tele-rehabilitation program by patients.

*Assessment of the physiotherapy rehabilitation program* Specifically, 93% of patients and 100% of their parents reported that the TRP helped them to correct and improve the home-exercise program. Parents expressed their tranquillity in terms of improving their children’s compliance, without having constant quarrels with them. There was a statistically significant reduction in the need to urge the patient to practice the home-exercise program and of the number of the required exhortations. Patient responses are illustrated in **Figure 7**.

## DISCUSSION

This is the first comprehensive physiotherapeutic tele-rehabilitation research in Greece and one of the very few, with interactive real-time videoconferencing, for patients with JIA, worldwide. It offers a new approach to physiotherapy for JIA patients in remote areas, balancing inequalities in health benefits. It assesses the influence of this intervention on the main goals of the treatment group (focus on pain and residue that affect the functionality, the patient’s QOL and the daily routine of the family) and on the current family status (internet, economic crisis). Most importantly, this study investigates how home intervention reduces the overall disease burden while encouraging the continuation of the home-exercise program (enhanced compliance).

This study also supports the basic concept of telemedicine and tele-rehabilitation, which is health provision to everyone. In particular, the TRP involved patients living in 12 different regions of Greece (8/15 live in a village and 7/15 in town), 13 live outside Thessaloniki.

Nowadays, Internet access in 68.1% of Greek households (according to the Hellenic Statistical Authority, ELSTAT 2015) data allowed us to use this means of intervention.^21^ Also, the data for the 13-17-year-old age group are impressive, with 98.8% using the internet (87% using it almost daily), while 3/5 children aged 7-12 years use a computer every day and 86% of them the Internet.^22^ These data explain the participants’ familiarization with the TRP and their wish to continue the program after the study. A study by Slater et al, which recorded the experiences and perceptions of young Australians with persistent musculoskeletal pain, regarding the role of digital technologies in supporting their health, also enhances this notion. As the study mentions, 99% of young people in Australia use the Internet (95% daily), which promotes the applicability of technological programs.^23,24^ There were no drop-outs in this study, all patients complied with the program and none showed evidence of disease deterioration (physician’s Vas remained <2). This suggests that the program is feasible and safe for our patients’ population. In contrast, in the study by Tarakci et al. regarding a home-based exercise program for patients with JIA (but not tele-rehabilitation), four patients dropped-out, one deteriorated, and the exported results pertained only to patients who had completed more than 75 % of the exercise program.^25^ A probable reason was the distance from the study’s centre and the training for the home-exercise program through brochures.

Pai and Mc Grady reported that the interaction of three parameters, ie ability, opportunity and motivation adapted to patient age and personality, can increase compliance and self-management of the disease. When a parameter is absent, self-management is hampered.^26,27^ Our intervention positively affected all these three parameters. The study provided training for the exercises and continuous guidance (ability), encouragement by the specialist to each patient, regardless of their residence (opportunity), and the targeting of small functional and feasible goals (motivation).

The program of exercises in this study was as short in duration as 25-30 minutes. Another factor for the efficacy of this TRP, apart from the program’s individuality, is the continuous supervision by a specialist. This is supported by Van den Berg et al., who found that an Internet-based intervention is more effective when the program is customised and implemented under supervision.^28^

JIA patients have limited functionality (stiffness, mobility limitation, muscle atrophy, coordination disorder and pathological pattern) as a consequence of arthritis. This can have a significant impact on the patient’s daily routine, with limitations on their activity, pain, and an overall effect on patient’s QOL.^29^ This study focused on the effect of a TRP on these parameters with statistically significant improvement. Moreover, the disease residues improved in all patients, who practiced a special home-exercise program, but a greater improvement was recorded in the TRG patients. The above results are consistent with the recommendations of the Ottawa panel in 2016 that include a home-exercise program as an essential part of the physiotherapeutic approach, in order to improve the QOL and functional capacity of JIA patients, without requiring “hands on” intervention.^30^ Similarly, Tarakci et al. showed that participation in a 12-week exercise program, four times a week, can lead to an improvement in the above-mentioned parameters.^25^ However, the authors noted the distance from the research centre and the exercise training through leaflets as restrictions in their study. These limitations are completely eliminated in our study. We excluded the distance-factor with the use of the internet and we applied an interactive and personalized monitoring of the exercise program, online. Thus, all patients completed the program.

The ability to implement the TRP as well as the satisfaction with the program was rated as excellent by both patients and parents. These results are in line with most of the published studies on interventions through the Internet for children or adults.^31–33^

Most participants, enjoying the program, showed an interest early on to continue the TRP after the end of study. This was underlined by the very high scores in patients and parents’ answers regarding the wish to continue the TRP, voicing the great help they received and the security they felt on how to apply the exercises.^34^

It must be taken into account that most of the study patients had the same physiotherapist, who performed the TRP and that all participants, had known her for a long time from their regular follow-ups at the Centre, trusting her as a person and professional. Navarro et al. emphasise that parents apply the exercise program more regularly when the physiotherapist builds a trusting relationship from the beginning, minimises their fears about their ability to implement the program, and helps them to integrate the exercises in the child’s daily routine.^35^

In the present study, interactive training and correction of the TRP was conducted at the time of its implementation. This may well explain the improved compliance and tranquillity in everyday life. It is certain that this should be a prerequisite or a safe guard for the implementation of such programs.

Given the timesaving and economic benefit of remote tele-rehabilitation programs, these interventions do not have to demonstrate superiority to face-to-face treatments, but equal results seem to be enough.^36^

A recent study by Hugle et al. on the prescribing of physiotherapy in patients with oligoarticular JIA, showed that physicians from Germany/Austria frequently recommended regular physiotherapy compared to Canadian doctors (85.6% vs. 13.9%), who often chose a home-exercise program after training (52.8% vs. 14.4% of German/Austrian doctors). Similar percentages were also recorded for patients with polyarticular JIA. This is because in Germany and Austria health insurance usually covers physiotherapy in JIA patients, while in Canada, services are often funded privately.^37^

Given that in our country the health system provides minimal physiotherapeutic support for JIA, tele-rehabilitation physiotherapy techniques that do not require “hands on” application provide a feasible and effective solution.

An important factor in all internet interventions is the healthcare providers’ training. A study by Lawford et al. across Australia explored the perceptions and willingness of 217 physiotherapists regarding the use of online models of exercise services for people with knee and/or hip osteo-arthritis. This study showed that while the physiotherapists had an overall positive perception of video tele-recovery compared to telephone care, they had little confidence in the use of interactive online technologies and reported no previous experience in remote rehabilitation. More than a third (37-45%) of the participants did not believe that the use of video technologies would be easy, or a safe way for patients to receive an exercise program.^4^

The main limitation of the present study is the relevant small sample size. The involvement of more patients requires more researchers, risking the uniformity in the services provision. In addition, it would need a longer time of intervention and familiarisation of the involved specialists with the paediatric rheumatology physiotherapy.

## CONCLUSIONS

This study introduces a new rehabilitation proposal to promote physiotherapy for JIA patients over the internet in addition to current physiotherapy sessions. Our study indicates that a TRP is feasible, applicable, and has a positive effect on JIA patients and their families. It provides an additional tool to the treatment group, which copes with the already well-known disease difficulties and requirements as well as the particularities of our country (economic crisis, uneven geographical distribution and limited organised paediatric Rheumatology centres, and inadequacies of the public health system). Specifically, significant improvement was noticed in the TRG patients compared to the CG patients regarding pain and QΟL, but also on the parameters related to the implementation of the home exercise program, (compliance, need for urging and number of exhortations, frequency of implementation).

Tele-physiotherapy also expands the patients’ choices regarding the home rehabilitation program, promoting equality in health care. At the same time, tele-physiotherapy provides health professionals with an additional incentive for specialisation.

Further studies are needed in order to establish the already available applications in paediatric tele-physiotherapy, to evaluate their efficacy and to develop new ones.

## APPENDIX

### JAMAR

The JAMAR questionnaire is a special questionnaire for multidimensional (total) assessment of the effects of JIA on the physical, psychological and social functioning, as perceived and recorded by the children themselves and their parents for ages 8 years and older and only parents for ages up to 8 years. It has been validated for the Greek population.^19,20^

Specifically, it concerns the following sectors:

- Functionality,
- Symptoms and course of the disease,
- Process and compliance in taking medication with recording of difficulties and possible side effects,
- School attendance and the child’s quality of life.

It also includes VAS (visual analogue scale) scales from 0–10 for:

- the pain
- the state of the disease (activity, remission) and
- the overall assessment of the impact of the disease on all areas of the child’s life.

In order to quantify patients’ functionality, their responses to the 15 “functional capacity assessment” questions were scored as follows: no difficulty=3 points, little difficulty=2 points, very difficult=1 point, cannot be performed=0 degrees. Then, the scores of each response for each patient were summed, resulting in a sum with a maximum value of 45 (excellent functioning) and a minimum of 0. Accordingly, in order to quantify patients’ quality of life, their responses to the 10 “functional capacity assessment” questions were scored as follows: never=3 points, sometimes=2 points, often=1 point, every day=0 points. Then, the scores of each response for each patient were summed, resulting in a sum with a maximum value of 30 (excellent quality of life) and a minimum value of 0.

### STUDY QUESTIONNAIRE

For the needs of this research, a new evaluation questionnaire was created for the patient and for the parent or guardian, in relation to the patient’s participation in a program of physical therapy sessions and in relation to the implementation of the special exercise program at home.

Specifically, it contained questions regarding:

- The regularity of the application of the exercises
- Compliance with the instructions
- Whether the exercises are done with or without supervision
- Whether or not the child himself knows the exercises but also the parent or guardian who participates as an assistant or as a supervisor of the implementation of the exercises
- The child’s weekly schedule of activities outside of school
- The child’s participation in sports activities and
- The assessment of the child’s compliance in the implementation of the exercise program at home, on a VAS type scale from 0–10.

After the end of the tele-rehabilitation program, the specific questionnaire also included evaluation questions of the program from the patients and parents of OTA, with scales 0–10, which related to:

- The possibility of implementing the tele-rehabilitation program
- Its offer in the following areas:
  1. Training the patient in the exercise program
  2. Self-management training
  3. Everyday life of the family
  4. Functionality and in the improvement of the remains of the disease
  5. Training of the parent or guardian in the exercise program and
  6. Effectiveness of the application of the exercises with the supervision of the physical therapist in relation to the application of the exercises without him.
- The assessment of the overall offer of the tele-rehabilitation program - degree of satisfaction, and
- The desire to continue its implementation, in the long term.

Also, in the new assessment questionnaire filled in by patients and parents of OTA after the end of the program, there was an assessment on a scale of 0-10 regarding whether or not corrections were made to the exercise program as a whole by the physical therapist, during of the video sessions and whether the initial response to the question “I know the exercises” actually corresponded.
